# Rheostatic Balance of Circadian Rhythm and Autophagy in Metabolism and Disease

**DOI:** 10.3389/fcell.2020.616434

**Published:** 2020-11-24

**Authors:** Xiang Wang, Zhijie Xu, Yuan Cai, Shuangshuang Zeng, Bi Peng, Xinxin Ren, Yuanliang Yan, Zhicheng Gong

**Affiliations:** ^1^Department of Pharmacy, Xiangya Hospital, Central South University, Changsha, China; ^2^Department of Pathology, Xiangya Hospital, Central South University, Changsha, China; ^3^Key Laboratory of Molecular Radiation Oncology of Hunan Province, Center for Molecular Medicine, Xiangya Hospital, Central South University, Changsha, China; ^4^National Clinical Research Center for Geriatric Disorders, Xiangya Hospital, Central South University, Changsha, China

**Keywords:** autophagy, circadian rhythm, mTOR, AMPK, diseases

## Abstract

Circadian rhythms are physical, behavioral and environmental cycles that respond primarily to light and dark, with a period of time of approximately 24 h. The most essential physiological functions of mammals are manifested in circadian rhythm patterns, including the sleep-wake cycle and nutrient and energy metabolism. Autophagy is a conserved biological process contributing to nutrient and cellular homeostasis. The factors affecting autophagy are numerous, such as diet, drugs, and aging. Recent studies have indicated that autophagy is activated rhythmically in a clock-dependent manner whether the organism is healthy or has certain diseases. In addition, autophagy can affect circadian rhythm by degrading circadian proteins. This review discusses the interaction and mechanisms between autophagy and circadian rhythm. Moreover, we introduce the molecules influencing both autophagy and circadian rhythm. We then discuss the drugs affecting the circadian rhythm of autophagy. Finally, we present the role of rhythmic autophagy in nutrient and energy metabolism and its significance in physiology and metabolic disease.

## Introduction

Autophagy is an intracellular degradative procedure that targets cytosolic components to lysosomes for degradation to maintain cellular homeostasis and provide substrates for energy generation (Farias et al., 2019; Santin-Marquez et al., 2019; Yan et al., 2019). The activation of autophagy frequently occurs in the context of nutrient shortages and other stresses (Jacomin et al., 2020; Martel et al., 2020). Cytoplasmic materials are first surrounded by vesicles, which eventually form double membrane structures called autophagosomes (Klionsky et al., 2014; Gou et al., 2020). The autophagosomes then fuse with lysosomes and create autolysosomes where the cargo is degraded and released as critical nutrients, such as fatty acids, back into the cytosol (Gatica et al., 2018; Li et al., 2020; Patra et al., 2020). Autophagy is capable of regulating many0020biochemical processes, including embryogenesis, development, antigen presentation, metabolism and infection removal (Bonam et al., 2020; Farhan et al., 2020; Wu and Nagy, 2020). Aberrant autophagy leads to various diseases, such as neurodegeneration, cancer, aging process, autoimmunity, and others (Choi et al., 2013; Bonam et al., 2018; Brattas et al., 2020; Devis-Jauregui et al., 2020; Mizushima and Levine, 2020; Oeing et al., 2020). At present, of the three known basic types of autophagy, chaperone-mediated autophagy, microautophagy and macroautophagy, most of our current knowledge is focused on macroautophagy (hereafter referred to as autophagy), which is the center of this review (Kaushik and Cuervo, 2018; Lescat et al., 2020; Schafer et al., 2020; Zheng et al., 2020). Additionally, studies have shown that autophagy is a dynamic process in the biological circadian rhythm, which is related to the degradation of cellular components and is driven by a series of autophagy-related proteins (ATGs) (Sachdeva and Thompson, 2008; Wesselborg and Stork, 2015; He Y. et al., 2016).

**FIGURE 1:**
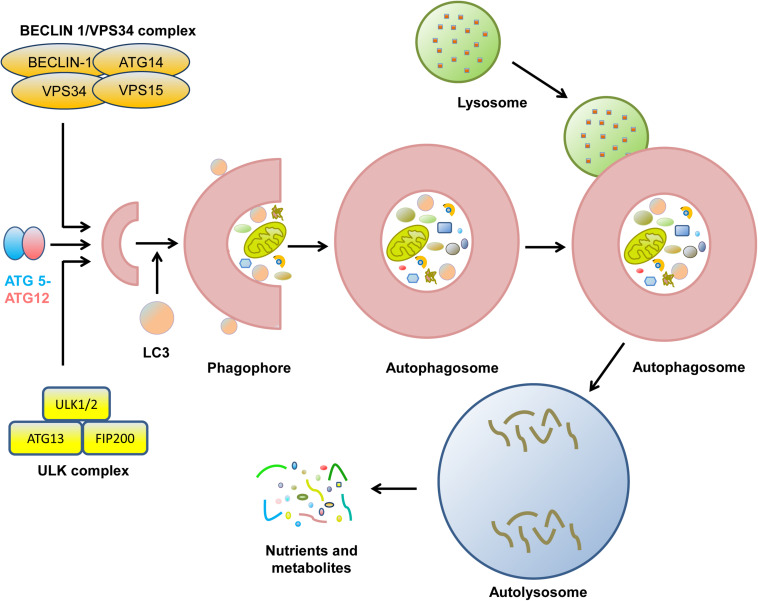
The biological process of cell autophagy. The initial stages of autophagy involve the nucleation, elongation, and maturation of a separation membrane, commonly known as a phagophore. Then, the formed phagophore unites to form the autophagosome. During the complex process, many protein complexes and autophagy-related proteins are sequentially involved. Eventually, autophagosome fuse with lysosome to form autolysosome, where the captured materials are eliminated. The final biomolecules are recycled back to the cytoplasm.

Autophagy is an evolutionarily conserved catabolic process containing five unique phases: initiation, vesicle nucleation, vesicle elongation, vesicle fusion, and cargo degradation (Figure 1; Levy et al., 2017). The initiation of autophagy is caused by nutrient deprivation, infection, oxidative stress, and other factors (Pavlinov et al., 2020). Vesicle nucleation is mediated by the activation of the Unc-51-like autophagy activating kinase (ULK1) complex, which is composed of ULK1/2, ATG13, ATG 101, and focal adhesion kinase-family interacting protein of 200 kDa (FIP200), which is suppressed by activation of mechanistic target of rapamycin (mTOR) (Petherick et al., 2015; Vahsen et al., 2020). Next, Bcl-2-interacting protein (BECLIN)-1 is phosphorylated by ULK1 and acts as a scaffold to form the class III phosphatidylinositol-3 kinase (PI3K) complex (Fracchiolla et al., 2020). During this process, the ultraviolet radiation resistance-associated gene proteins ATG14 and p150 bind to BECLIN-1 to facilitate its interaction with vacuolar protein sorting protein 34 (VPS34) and phagophore formation (Boukhalfa et al., 2020; Wu M. Y. et al., 2020). Vesicle elongation is induced by two ubiquitin-like conjugating systems, ATG12-ATG5 and microtubule-associated protein 1A/1B-light chain 3-II (LC3-II), together with p62 and other molecules, resulting in the formation of a compartment called the autophagosome (Lystad et al., 2019; Yan et al., 2019). Finally, syntaxin 17 promotes the fusion of the autophagosome and the lysosome for autolysosome formation, with the degradation of cargo when the pH is lowered (Galluzzi et al., 2017; Levy et al., 2017). During the process of autophagy, many molecules, such as mTOR and ATGs, display robust circadian rhythms (Maiese, 2017; Kim et al., 2019).

Circadian rhythms are daily predictable changes in physiology, behavior and environment with a period of time of approximately 24 h (Greco and Sassone-Corsi, 2020; Kim and Lazar, 2020). They exist on every biological scale, from macroscopic aspects such as sleep-wake cycles to microcosmic phenomena such as the rhythmic abundance of biomolecules (Ryzhikov et al., 2019b; Tan X. et al., 2019; Van Dyck and Casaer, 2019). In mammals, the daily changes are mediated by the hypothalamic master clock located in the suprachiasmatic nucleus (SCN) and by clock oscillations in peripheral tissues, which are synchronized by light and feeding time, respectively (Dreyer et al., 2019; Paul et al., 2020). The pacemakers are self-sustaining oscillators in the brain and peripheral tissues that synchronize their downstream transcriptional output (Carmo-Silva and Cavadas, 2017; Skene et al., 2018). A growing number of circadian-clock-controlled physiological processes exhibit daily oscillations, such as autophagy, whereas the dysfunction of the circadian system induced by shift work, for example, can increase the risk of many diseases, such as cancer (Rijo-Ferreira and Takahashi, 2019; Ryzhikov et al., 2019a).

**FIGURE 2:**
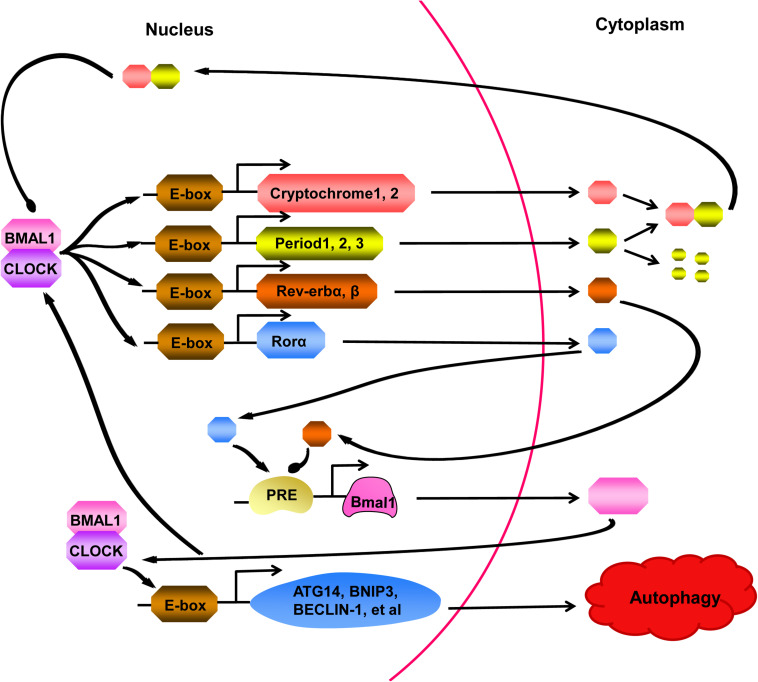
A series of cell processes are controlled by the circadian clock, which is activated by BMAL1:CLOCK heterodimer. The BMAL1:CLOCK complex binds to E-boxes in the promoters of target molecules, activating transcription of the Cryptochrome (CRY) genes, Period (PER) genes, Rev-erb genes, and Ror genes. PER proteins are degraded in the cytoplasm by the proteasome, however, binding to CRY prevents PER from degrading. The complex formed by PER and CRY can return to nucleus and suppressed activity of the BMAL1:CLOCK heterodimer. In a parallel arm of the transcriptional loop, Rev-erb and Ror proteins can compete for combining to the Bmal1 promoter, showing us another layer of regulation to cycling of the clock. Moreover, BMAL1:CLOCK complex can also activate the transcription of genes, participating in regulating many cell processes, such as ATG14, BNIP3, BECLIN-1. At present, studies have confirmed that autophagy can be regulated by the clock.

Recent studies have discovered that “clock genes” are essential for circadian rhythm generation (Figure 2; Kim and Lazar, 2020). In the clock gene family, members of the basic helix-loop-helix-PAS transcription factor family, BMAL1 and CLOCK, form heterodimers, which bind to E-boxes in the promoters of target genes to activate the expression of the Period (PER1, PER2, and PER3) and Cryptochrome (CRY1 and CRY2) genes (Jahanban-Esfahlan et al., 2018; Gabryelska et al., 2020). PER proteins are degraded in the cytoplasm by the proteasome, while PER:CRY heterodimers can translocate to the nucleus, preventing PER degradation, to suppress and block the activity of the BMAL1:CLOCK complex (Rabinovich-Nikitin et al., 2019). In a parallel arm of the transcriptional loop, the BMAL1:CLOCK heterodimer activates the transcription of orphan nuclear receptors REV-ERBs (REV-ERBα and REV-ERBβ) and RORs (RORα, RORβ, and RORγ) (Sulli et al., 2018). REV-ERBs and RORs can compete for binding to the Bmal1 promoter to activate and inhibit gene transcription effects, respectively, resulting in the circadian oscillation of Bmal1 (Maiese, 2017). In addition to regulating clock components, BMAL1:CLOCK also activates the transcription of genes involved in regulating autophagy, for example (Dong et al., 2016; Scotton et al., 2016; Maiese, 2017).

Here, we provide a general overview of the circadian rhythm of autophagy and discuss the potential mechanisms between circadian and autophagy. Moreover, we investigated the molecules and drugs affecting circadian rhythm and autophagy. Finally, we highlight the functions of the circadian rhythm of autophagy in biological processes and diseases.

### Overview of the Circadian Rhythm of Autophagy

The first evidence for the connection between autophagy and circadian regulation was discovered in the early 1970s. A series of electron microscopy studies performed by Pfeifer and colleagues proved that the number of autophagic vacuoles varies on the basis of the time of day in several rat tissues, including hepatocytes, cardiomyocytes, proximal tubules of kidney, pancreatic acinar cells, and the inner segment of retina rod cells (Ma and Lin, 2012; Czaja et al., 2013; Li and Lin, 2015). In addition, the volume and numeric density of autophagic vacuoles in the heart were discovered to maintain a diurnal pattern, which peak at the late-night phase and decrease to lower levels in the early-dark period (Ma et al., 2011). Apart from heart tissue, recent work demonstrated that autophagy activity also presents a robust diurnal rhythm in skeletal muscle and liver through the combination of more specific autophagy markers and flux measurements (Ma et al., 2012). LC3-II, a molecular marker for autophagy induction, can be used to evaluate autophagy flux by its degradation rate. This measurement indicated that the rate of LC3-I to LC3-II conversion in the liver obviously peaks during the noon phase and later declines toward the dark phase (Czaja et al., 2013). In addition, autophagy undergoes rhythmic variation consistent with the circadian pattern of feeding in adult mammals. Studies have found that the number of autophagic vacuoles reaching the maximum before the initiation of feeding and the minimal number of autophagic vacuoles occurring after the animal has begun to feed (Mizushima and Murphy, 2020; Packer, 2020). These findings showed that autophagy, which is influenced by a series of factors, such as circulating hormones, may be inhibited under energy-rich conditions and be induced during starved conditions (Sachdeva and Thompson, 2008). Moreover, in the liver, the cyclic activation of autophagy flux is related to the rhythmic expression of autophagy genes (Li and Lin, 2015). In general, these studies confirmed that peripheral regulated circadian-dependent autophagy is manipulated in several organ systems and possibly plays a critical role in tissue and organ repair. This may also provide reasons for the theory that sleep hours are essential for daily physiological processes and tissue maintenance.

Additionally, the circadian regulation of autophagy was not only found in mammals but also discovered in eukaryotic cells, which contain several oscillating genes in the autophagy pathway (Kijak and Pyza, 2017). In yeast, microarray studies indicated that more than 50% of the yeast genome is loop-controlled in the period of metabolic restriction (Tu et al., 2005). Furthermore, the regulation of autophagy genes seems to follow a particular temporal expression pattern with a decrease in metabolic functions. This finding confirmed the point that cell homeostasis and cell death events take place simultaneously, which is greatly conserved even in a simple eukaryotic cell (Rabinovich-Nikitin et al., 2019).

### Circadian Rhythm Regulation of Autophagy

The autophagy activities and the number of autophagic vacuoles have been found to vary during the day in many tissues. Several genes and pathways involved in autophagy are present in a rhythmical manner during diurnal variation (Table 1).

**TABLE 1:** Genes and pathways involved in the circadian rhythm regulation of autophagy.

**Gene/Pathway**	**Target molecular**	**Function**	**Regulation**	**Organism/cell culture**	**References**
C/EBPβ	ULK1, LC3B, BNIP3 and GAPRAPL1	Autophagy	Up	Mice	Ma and Lin, 2012
AMPK	ULK1, TSC1/TSC2 and mTOR complex 1	Autophagy	Up	HeLa cells	Shang et al., 2011
PGC-1α/PGC-1β	ULK1, BNIP3, FIP200, GAPRAPL1, ATG2A, ATG16L1 and ATP6V0A2	Autophagy	Up	Mice	Li and Lin, 2015
TFEB/TFE3	Rev-erbα	Autophagy	Up	Mouse	Pastore et al., 2019
FoxO3	LC3B, BNIP3, BNIP3L and GAPRAPL1	Autophagy	Up	Mice	Mammucari et al., 2007; Ruan et al., 2020
SREBF2	PNPLA8	Autophagy	Up	Mice	Oishi et al., 2015; Kim et al., 2016
HSF-1	ATG10 and ATG18	Autophagy	Up	Tomato	Wang et al., 2015; Li et al., 2016
HSF-1	LGG-1/ATG8, LGG-2/ATG8 and EPG-9	Autophagy	Down	*C. elegans*	Barna et al., 2018
ACBP3	ATG8	Autophagy	Down	*C. elegans*	Xiao and Chye, 2010

CCAAT/enhancer binding protein β (C/EBPβ) is a basic leucine zipper transcription factor that is critical for the activation of autophagy in response to starvation as well as during light/dark cycles (Xu et al., 2018). C/EBPβ can directly bind to the promoters of autophagy genes and induce their transcription (Barakat et al., 2016). At present, C/EBPβ is emerging as a significant factor involved in rhythmic autophagy gene expression, such as ULK1, microtubule-associated protein 1A/1B-light chain 3B (LC3B), BCL2/adenovirus E1B interacting protein 3 (BNIP3) and GABA (A) receptor-associated protein like 1 (GABARAPL1), which are expressed in an oscillating manner and are mediated by circadian and nutritional signals (Ma and Lin, 2012). In an *in vivo* study, Ma et al. found that adenoviral-mediated RNAi knockdown of C/EBPβ eliminated circadian-regulated autophagy. Furthermore, these authors also discovered altered C/EBPβ levels, disrupted rhythmic regulation of autophagy and decreased autophagic gene expression in liver-specific BMAL1 knockout mice (Ma et al., 2011). Interestingly, in this study, low autophagic flux occurred in line with feeding with the onset of the dark phase, while 24-h starvation did not influence the rhythmicity of autophagy gene expression. The results showed an association between the cyclic regulation of autophagy and the dependence of autophagic rhythm on nutritional signals. This finding indicated that the zeitgebers of the circadian rhythm can directly influence the temporal and spatial expression of autophagic genes.

Another bridge that functionally connects autophagy and circadian rhythm is the adenosine monophosphate-activated protein kinase (AMPK) pathway. AMPK is a well-known energy-sensing kinase that participates in various catabolic and anabolic processes, including oxidative metabolism, glucose uptake, and nutrient biosynthesis (Gonzalez et al., 2020). In addition, autophagy is negatively regulated by the mTOR signaling pathway and by a downstream cascade of Atgs, such as BECLIN-1, LC3, ATG1, and ATG5 (Mizushima et al., 2008). Recently, studies have shown that AMPK can induce autophagy through dual mechanisms, including the direct activation and the abolishment of inhibitory effects of mTOR complex 1 on ULK1 through activation of the hamartin (tuberous sclerosis 1)/tuberin (tuberous sclerosis 2) (TSC1/TSC2) complex (Kim et al., 2011; Shang et al., 2011). Interestingly, both AMPK and mTOR show rhythmic regulation. Lamia et al. found that AMPK activity and nuclear localization, which showed a cyclic rhythm, were negatively related to CRY1 nuclear expression in mouse hepatocytes (Lamia et al., 2009). In addition, activation of AMPK unbalanced CRY proteins and normal rhythmic oscillations. Destruction of AMPK pathways significantly impaired peripheral clock activity in mice (Lee and Kim, 2013). Thus, AMPK provides another direction for the rhythmic regulation of autophagy based on its ability to control the cyclic expression of mTOR through regulation of CRY1.

The peroxisome proliferator–activated receptor gamma coactivator 1 (PGC-1) family of transcriptional coactivators includes PGC-1α and PGC-1β (Piccinin et al., 2018). PGC-1α and PGC-1β can activate the expression of mitochondrial gene programs encoded by both nuclear and mitochondrial genomes in many cell types (Nierenberg et al., 2018). In addition, studies have verified that the expression of PGC-1α and PGC-1β presents robust circadian rhythms in the liver and skeletal muscle. For PGC-1α, the association with circadian gene expression indicated that PGC-1α might regulate mitochondrial turnover in a circadian rhythm manner (Sonoda et al., 2007). Moreover, mice lacking PGC-1α exhibit aberrant light/dark cycles (Liu et al., 2007). Therefore, the interaction between PGC-1α and the circadian system might suggest another connection between circadian rhythm and autophagy. PGC-1β was discovered to physically connect with C/EBPβ in transiently transfected 293T cells, which indicated that it might be a C/EBPβ transcriptional coactivator (Li and Lin, 2015). More importantly, adenoviral-mediated PGC-1β overexpression was shown to promote the expression of autophagy genes regulated by C/EBPβ in hepatocytes, including ULK1, BNIP3, FIP200, GABARAPL1, ATG2A, ATG16L1, and ATPase H^+^ transporting V0 subunit a2 (ATP6V0A2) (Li and Lin, 2015). These results illustrated that PGC-1β might function as a coactivator for C/EBPβ to facilitate the expression of autophagy and strengthen autophagy activity. Thus, it is possible that the rhythmic activation of PGC-1β and C/EBPβ might regulate circadian signaling to activate autophagy rhythmically.

The MiT-TFE transcription factors TFEB and TFE3 are the primary regulators of autophagy, lysosomal biogenesis and lysosomal exocytosis by activating the expression of many genes involved in these processes (Annunziata et al., 2019; Yang et al., 2020). Recent studies have found that TFEB and TFE3 are activated in a circadian manner and promote the expression of Rev-erbα (Nr1d1), a transcription inhibitor component of the main clock machinery that controls autophagy-related gene expression, such as BECLIN-1, BNIP3, ATG5, ATG7, and ULK1, however, their depletion damages Rev-erbα expression and oscillation (Pastore et al., 2019). In addition, TFEB/TFE3 and Rev-erbα bind the common promoter regions, indicating that they can induce the rhythmic expression of genes involved in autophagy (Pastore and Ballabio, 2019). Therefore, this study provides a novel mechanism by which the dynamic balance between TFEB/TFE3 and Rev-erbα can be used to regulate the rhythmic oscillation of autophagy.

Moreover, several other transcription factors, including forkhead transcription factor O3 (FoxO3), sterol regulatory element binding transcription factor 2 (SREBF2), heat shock factor 1 (HSF-1) and acyl-CoA-binding protein 3 (ACBP3), are involved in the circadian rhythm regulation of autophagy. FoxO3, a regulator of autophagy, has been shown to induce the expression of autophagy genes, including LC3B, BNIP3, BCL2 interacting protein 3 like (BNIP3L), and GABARAPL1 (Mammucari et al., 2007; Ruan et al., 2020). SREBF2 can increase autophagy in hepatocytes of high-fat diet-fed mice by directly activating the expression of the patatin-like phospholipase domain-containing enzyme (PNPLA8) gene, which associates with autophagosomes, in a circadian manner (Oishi et al., 2015; Kim et al., 2016). HSF-1 displays a circadian rhythm, inducing and inhibiting autophagy by acting on different autophagy-related genes under certain cellular conditions (Wang et al., 2015; Li et al., 2016; Barna et al., 2018). The overexpression of ACBP3 reinforces the degradation of ATG8 and obstructed autophagosome formation (Xiao and Chye, 2010). A previous study found that the circadian regulation of ACBP3, which is upregulated in darkness but suppressed by light, is mediated by the *cis*-responsive elements DNA-binding with one finger box (Dof) and GT-1 (Zheng et al., 2012). Furthermore, autophagy-related genes and mTOR also display robust circadian rhythms (Rotter and Rothermel, 2012). For example, the expression of the ATG14 gene presents a circadian rhythm, which is controlled by Clock/Bmal1, the core clock component (Xiong et al., 2012; Jenwitheesuk et al., 2014). Therefore, understanding these molecules, an important aspect of the autophagy rhythm, is essential for further exploring the mechanisms of circadian regulation of autophagy.

### Regulation of Circadian Rhythm by Autophagy

Circadian rhythm can regulate autophagy and be regulated by autophagy. Jeong et al. (2015) first found evidence for the autophagic regulation of a core clock component. Pharmacological and molecular studies have shown the stabilization of BMAL1 by CLOCKΔ19 through the mechanism of attenuating the autophagic degradation of Bmal1 in Clk/ + mice. This investigation presented a novel style of clock regulation by autophagy. Another study showed that autophagy leads to the degradation of CRY1, which binds to LC3 via its LC3-interacting region (LIR) motifs, in the liver within a temporal window when rodents generally eat less and live on gluconeogenesis (Toledo et al., 2018). The livers were demonstrated to show enhanced CRY1 levels and reduced blood glucose levels in ATG7-deficient mice, which was rescued by reducing the hepatic CRY1 content. This phenomenon indicated that the autophagic degradation of CRY1 functions to maintain blood glucose levels during decreased feeding. This study also showed that PER2 might be a cargo adapter that promotes the interaction of CRY1 with LC3. Moreover, in a study on the mechanism of ferroptosis, Liu et al. discovered that autophagy facilitates ferroptotic cell death via the selective degradation of ARNTL/BMAL1 (aryl hydrocarbon receptor nuclear translocator-like), a vital circadian clock regulator, through the cargo receptor SQSTM1/p62 (Liu et al., 2019). Thus, these studies suggest that autophagy and circadian rhythm are reciprocally regulated.

### Molecular Regulation of Both Autophagy and Circadian Rhythm

There are several molecules that regulate both autophagy and circadian rhythm (Table 2). For example, casein kinase 1α (CK1α), belonging to the CK1 family of proteins, exhibits dual functions in autophagy regulation (Cai et al., 2018; Hermanova and Carracedo, 2018). Suppression CK1α by D4476, a CK1 inhibitor, or siRNA-mediated knockdown of CK1α leaded to the inhibition of mTOR signaling, and activation of autophagy (Gao et al., 2011; Zhao et al., 2011). CK1α could suppress p53 downstream of MDM2-mediated autophagy in acute myeloid leukemia (Xu et al., 2020). However, the overexpression of CK1α has been shown to convectively activate autophagic flux in non-small cell lung cancer (NSCLC) through the PTEN/AKT/FOXO3A/ATG7 axis (Jiang et al., 2018). In addition, CK1α-mediated phosphorylation facilitates the degradation of PER1, indicating an effect on circadian rhythm (Lam et al., 2018). Melatonin, N-acetyl-5-methoxytryptamine, is produced by the pineal gland and various other tissues (Luo et al., 2019). The regulation of autophagy by melatonin is depending on physiological status and diseases. Under the state of aging, melatonin induces autophagy by decreasing the mitochondrial membrane rigidity, however, as for virus infection and high fructose consumption, melatonin functions as an inhibitor of autophagy via the antioxidant-mediated effect and ER stress-mediated effect (Garcia et al., 2011; San-Miguel et al., 2014; Xie et al., 2015; Lin C. et al., 2016; Bermejo-Millo et al., 2018). In addition, in some diseases, such as cancers, neurodegeneration and obesity, melatonin plays a dual role in autophagy through regulating the ROS/MST1 and PI3K/Akt/mTOR signaling pathways (Zheng et al., 2014; Yoo et al., 2016; Shen et al., 2018; Shi et al., 2018; Boga et al., 2019). Moreover, melatonin can upregulate the expression of CLOCK and PER2 proteins in human prostate cancer cells, showing an influence on circadian rhythm (Jung-Hynes et al., 2010). Silent mating type information regulation 2 homolog 1 (Saccharomyces cerevisiae) (SIRT1), a member of the sirtuin family, can promote autophagy by blocking mTOR or activating AMPK (Wu Y. et al., 2020). Furthermore, SIRT1 enables deacetylation of histone BMAL1, PER, and CRY and thus regulates the transcription of circadian proteins and hence the cycle (Hirayama et al., 2007; Chung et al., 2010; Maiese, 2018). HSF1 exhibits dual functions in autophagy, as mentioned above, and can also induce the synchronization of the circadian clock by regulating PER2 protein directly (Tamaru et al., 2011; Li et al., 2016; Watanabe et al., 2017). Therefore, these molecules play an essential role in the regulation of rhythmic autophagy and can be used as targets for the clinical treatment of diseases.

**TABLE 2:** Molecular regulation of both autophagy and circadian rhythm.

**Molecule**	**Target molecule/pathway**	**Function**	**Regulation**	**Organism/Cell culture**	**References**
CK1α	p53/AMPK/mTOR	Autophagy	Down	HL-60, HEL	Xu et al., 2020
CK1α	PTEN/AKT/FOXO3A/Atg7	Autophagy	Up	NSCLC	Jiang et al., 2018
CK1α	PER1	Circadian rhythm	Up	Drosophila	Lam et al., 2018
Melatonin	mTOR/Akt	Autophagy	Up	Cal-27 and SCC-9	Shen et al., 2018
Melatonin	ROS-MST1	Autophagy	Down	Rat	Shi et al., 2018
Melatonin	PI3K/Akt/mTOR	Autophagy	Down	Rat	Zheng et al., 2014
Melatonin	CLOCK and PER2	Circadian rhythm	Up	LNCaP, 22Rν1, DU145, and PC3	Jung-Hynes et al., 2010
SIRT1	mTOR	Autophagy	Up	Mice	Wu Y. et al., 2020
SIRT1	BMAL1, PER, and CRY	Circadian rhythm	Down	Mouse	Hirayama et al., 2007; Chung et al., 2010; Maiese, 2018
HSF1	SQSMT1/p62	Autophagy	Up	HeLa	Watanabe et al., 2017
HSF1	LGG-1, LGG-2, ATG2, ATG9, ATG11, and ATG18	Autophagy	Down	*C. elegans*	Li et al., 2016
HSF1	PER2	Circadian rhythm	Up	Mouse	Tamaru et al., 2011

### Drugs Affecting the Circadian Rhythm of Autophagy

In addition to certain molecules influencing rhythmic autophagy, some drugs can also exert similar effects (Table 3). For example, rapamycin, already used in clinical practice, is a potent regulator of autophagy (Alvers et al., 2009; Pulakat and Chen, 2020). Chloroquine and Lys05 are clinically relevant lysosomotropic agents that inhibit autophagy (Baquero et al., 2019; Erkisa et al., 2020). The compound SR8278 is the first REV-ERB antagonist, although its pharmacokinetic properties limit its pharmacological uses (Kojetin et al., 2011). A recent study found that the compound ARN5187 has a dual inhibitory effect on both autophagy and REV-ERB in BT-474 cells (De Mei et al., 2015). ARN5187 was demonstrated to have the ability to block lysosomal function, disrupt the autophagy process in the late stage and decrease breast cancer cell viability, which showed that it is an autophagy suppressor. ARN5187 was also shown to repress REV-ERB-mediated transcription regulation. Moreover, ARN5187 was demonstrated to be more cytotoxic than chloroquine, and REV-ERB inhibitors were shown to be able to enhance the cytotoxicity of chloroquine in BT-474 cells. In addition, research has showed that folic acid deficiency can enhance the activity of autophagy in HT-22 hippocampal neuron cells *in vitro*, accompanied by the induction of the expression of autophagy- and circadian-related genes, such as ATG12, ATG13, and PER2, through the glucocorticoid receptor-mediated pathway (Sun et al., 2016). This research suggested that folic acid might be involved in the regulation of rhythmic autophagy. Apart from these results glucocorticoids have been reported to decrease autophagic activity in the placenta and bone, whereas they are activated in muscle and lymphocytes (Harr et al., 2010; Troncoso et al., 2014; He B. et al., 2016; Lin N. Y. et al., 2016). Morphine was shown to increase the mRNA expression levels of autophagy-related genes, including ATG3, ATG5, ATG7, and ATG12, in C6 cells (Feng et al., 2013). Autophagy can be regulated during doxorubicin-induced cardiotoxicity (Kobayashi et al., 2010; Sishi et al., 2013). In summary, these studies demonstrated the significance of rhythmic autophagy in organisms and suggest that this process could be used as a target for the clinical therapy of diseases.

**TABLE 3:** Drugs affecting the circadian rhythm of autophagy.

**Year**	**Author**	**Drug**	**Function**	**Regulation**	**Organism/Cell culture**	**References**
2009	Alvers et al.	Rapamycin	Autophagy	Up	*C. elegans*	Alvers et al., 2009
2020	Erkisa et al.	Chloroquine	Autophagy	Down	PC-3	Erkisa et al., 2020
2019	Baquero et al.	Lys05	Autophagy	Down	HT-29	Baquero et al., 2019
2011	Kojetin et al.	SR8278	Circadian rhythm	Down	HepG2	Kojetin et al., 2011
2015	Mei et al.	ARN5187	Autophagy and circadian rhythm	Down	BT-474	De Mei et al., 2015
2016	Sun et al.	Folic acid (deficiency)	Autophagy	Up	HT-22	Sun et al., 2016
2015	Lin et al.	Glucocorticoids	Autophagy	Down	MC3T3-E1	Lin N. Y. et al., 2016
2016	He et al.	Glucocorticoids	Autophagy	Down	BeWo	He B. et al., 2016
2014	Troncoso et al.	Glucocorticoids	Autophagy	Up	L6	Troncoso et al., 2014
2010	Harr et al.	Glucocorticoids	Autophagy	Up	CEMC7	Harr et al., 2010
2013	Feng et al.	Morphine	Autophagy	Up	C6	Feng et al., 2013
2010	Kobayashi e al.	Doxorubicin	Autophagy	Up	Neonatal rat cardiomyocytes	Kobayashi et al., 2010
2013	Sishi et al.	Doxorubicin	Autophagy	Down	H9C2	Sishi et al., 2013

### The Applications of Circadian Rhythm of Autophagy in Organisms and Diseases

Rhythmic autophagy induction enables the maintenance of energy and nutrient homeostasis, the implementation of temporal compartmentalization of tissue metabolism and remodeling of proteomes and organelles throughout the light/dark and feeding cycles (Solanas et al., 2017; Mazzoccoli et al., 2018; Stockman et al., 2018). The concentrations of plasma amino acids and metabolites show intense circadian rhythms, which are mediated by autophagy in part. In addition, these nutrients can be used for the biosynthesis of essential macromolecules in nutrient-limited periods and can also enter systemic circulation for energy homeostasis in organisms (Ma and Lin, 2012). The expression of genes participating in glucose metabolism, cholesterol biosynthesis, *de novo* lipogenesis and fatty acid β-oxidation greatly oscillate in the liver, indicating that the coordination of circadian patterns of metabolic cycles with rhythmic autophagy has the ability to optimize nutrient storage and fuel oxidation (Tarquini and Mazzoccoli, 2017; Roohbakhsh et al., 2018).

Moreover, a comprehensive understanding of the associations between autophagy and circadian rhythm has significant impacts on human diseases (Kondratova and Kondratov, 2012; Esterline et al., 2018). For example, autophagy is involved in the regulation of tumor development and progression at both the cellular and organismal levels, a condition also impacted by circadian rhythm (Czaja et al., 2013; Chok et al., 2019). Autophagy has been shown to be involved in aging, a process related to circadian oscillation, based on the finding that the amassing of neural aggregates observed in aging is associated with a decrease in the autophagy pathway (Blagosklonny et al., 2010; Jirakkakul et al., 2018). The dysfunction of circadian rhythm is also connected to age-related neurodegenerative disorders, such as Alzheimer’s disease, in which autophagy plays a part in the pathogenesis and progression of disease (Chen et al., 2015; Doktor et al., 2019). The absence of the PER1 protein in the hippocampus might exacerbate the pathology of cerebral ischemia due to depressed autophagy (Maiese, 2017). Additionally, other diseases, such as osteoporosis, obesity, diabetes and cardiovascular disease, are also influenced by both autophagy and circadian rhythm (Blaney Davidson et al., 2017; McGinnis et al., 2017).

## Discussion and Conclusion

Interference with the circadian rhythm of autophagy has been demonstrated to result in many disorders in organisms (Rozman, 2018; Sarker and Franks, 2018; Dong et al., 2019). Therefore, the reasons for the diseases induced by rhythmic autophagy dysfunction are discussed here. Rhythmic autophagy is capable of removing damaged materials from cells regularly, and a deficiency in this process can lead to the accumulation of abnormal cellular components, which finally induce cells and whole organisms to undergo pathological conditions (Li et al., 2020; Wu and Nagy, 2020). Autophagy might be activated to eliminate damaged organelles, proteins and lipids after oxidative phases of metabolism (Pacheco et al., 2020). The dysfunction of rhythmic autophagy might cause misfolded proteins to gather in cells, which can lead to alteration in membrane permeability, the generation of reactive oxygen species, and the disruption of mitochondria and DNA, facilitating metabolic diseases, neurodegeneration and aging (Sachdeva and Thompson, 2008; Abdrakhmanov et al., 2020; Gu et al., 2020). The circadian rhythm of autophagy regulation is considered to limit the accumulation of dysfunctional cell components, which are the core constituents of the pathogenesis of each of these disorders.

In addition, autophagy plays an important role in the development, organization and functions of the immune system, including cell survival, cell-autonomous defense and regulation of complex multicellular immune responses (Keller et al., 2020). For example, autophagy is necessary for keeping T cell survival, development and functional integrity under the circumstance of activating, however, autophagy deficiency in Treg cells will result in defective Treg function (Wei et al., 2016; Tan P. et al., 2019). As mention above, autophagy present circadian rhythm in many tissues and light/dark phase can influence its activity. Therefore, we speculate that light phase and less dark phase or vice-versa may have effects on autoimmunity by affecting autophagy, although it has not been reported yet. Above all, maintain the circadian rhythm of autophagy is essential for the construction of immune system.

Thus, a comprehensive study of the relationship between autophagy and circadian rhythm will allow us to better understand multiple disease processes, including cancer, neurodegeneration, aging, and metabolic disorders, all of which have been associated either directly or indirectly with both autophagy and circadian-controlled genes. The understanding of the mechanisms of the interaction between autophagy and circadian rhythm provides insights into the clinical treatment of diseases.

## Author Contributions

ZG, YY, XW, and YC collected the related manuscript. ZX, XW, and XR drafted and wrote the manuscript. ZX, XW, SZ, BP and XR revised the manuscript. All authors have read and approved the final manuscript.

## Conflict of Interest

The authors declare that the research was conducted in the absence of any commercial or financial relationships that could be construed as a potential conflict of interest.

## Abbreviations

ATGs: Autophagy-related proteins
ULK1: Unc-51-like autophagy activating kinase
Mtor: Mechanistic target of rapamycin
PI3K: Phosphatidylinositol-3 kinase
FIP200: Focal adhesion kinase-family interacting protein of 200 kDa
VPS34: Vacuolar protein sorting protein 34
LC3-II: Microtubule-associated protein 1A/1B-light chain 3-II
LC3B: Microtubule-associated protein 1A/1B-light chain 3B
BNIP3: BCL2/adenovirus E1B interacting protein 3
GABARAPL1: GABA (A) receptor-associated protein like 1
ATP6V0A2: ATPase H^+^ transporting V0 subunit a2
SCN: Suprachiasmatic nucleus
PER: Period
CRY: Cryptochrome
C/EBPβ: CCAAT/enhancer binding protein β
AMPK: Adenosine monophosphate-activated protein kinase
BNIP3L: BCL2 interacting protein 3 like
PGC-1: Peroxisome proliferator–activated receptor gamma coactivator 1
FoxO3: Forkhead transcription factor O3
SREBF2: Sterol regulatory element binding transcription factor 2
HSF-1: Heat shock factor 1
ACBP3: Acyl-CoA-binding protein 3
PNPLA8: Patatin-like phospholipase domain-containing enzyme
Dof: DNA-binding with one finger box
LIR: LC3-interacting region
CK1α: Casein kinase 1 α
NSCLC: Non-small cell lung cancer
SIRT1: Silent mating type information regulation 2 homolog 1.
